# Entropy Analysis of RR-Time Series From Stress Tests

**DOI:** 10.3389/fphys.2020.00981

**Published:** 2020-08-12

**Authors:** Eric E. Solís-Montufar, Gonzalo Gálvez-Coyt, Alejandro Muñoz-Diosdado

**Affiliations:** ^1^Unidad Profesional Interdisciplinaria de Biotecnología, Instituto Politécnico Nacional, Mexico City, Mexico; ^2^Centro de Investigación en Computación, Instituto Politécnico Nacional, Mexico City, Mexico

**Keywords:** exercise, stress test, heart rate variability, tachograms, entropy, complexity, physical conditioning

## Abstract

The RR-interval time series or tachograms obtained from electrocardiograms have been widely studied since they reflect the cardiac variability, and this is an indicative of the health status of a person. The tachogram can be seen as a highly non-linear and complex time series, and therefore, should be analyzed with non-linear techniques. In this work, several entropy measures, Sample Entropy (SampEn), Approximate Entropy (ApEn), and Fuzzy Entropy (FuzzyEn) are used as a measure of heart rate variability (HRV). Tachograms belonging to thirty-nine subjects were obtained from a cardiac stress test consisting of a rest period followed by a period of moderate physical activity. Subjects are grouped according to their physical activity using the IPAQ sedentary and active questionnaire, we work with youth and middle-aged adults. The entropy measures for each group show that for the sedentary subjects the values are high at rest and decrease appreciably with moderate physical activity, This happens for both young and middle-aged adults. These results are highly reproducible. In the case of the subjects that exercise regularly, an increase in entropy is observed or they tend to retain the entropy value that they had at rest. It seems that there is a possible correlation between the physical condition of a person with the increase or decrease in entropy during moderate physical activity with respect to the entropy at rest. It was also observed that entropy during longer physical activity tests tends to decrease as fatigue accumulates, but this decrease is small compared to the change that occurs when going from rest to physical activity.

## Introduction

The variability of human heart rate (HRV), is obtained by measuring the beat-to-beat changes in the duration of the RR interval of the electrocardiogram (ECG), is the result of the combination of different physiological control systems, which operate on different scales temporary and that allow the functioning of the body to adapt to physical, environmental or other changes. Such fluctuations have been represented as a superimposition of rhythms, which contribute to the neuroautonomic modulation of the heart rhythm in healthy conditions, and are altered by a wide variety of disease states. In fact, there is a consensus among the scientific community that the long-term RR interval time series are non-linear and multifractal and that the HRV scale behavior is altered with aging, during physical exercise and in pathological conditions such as for example, myocardial infarction (Iyengar et al., 1996; Ivanov et al., 1999a; Huikuri et al., 2000; Bernaola-Galván et al., 2017; Gómez-Extremera et al., 2018; Faes et al., 2019). It is also widely accepted that the evaluation of HRV on different time scales has allowed us to give a quite satisfactory explanation of the short-term mechanisms underlying cardiovascular control (Malliani et al., 1991; Cohen and Taylor, 2002; Xiong et al., 2017). When heart rate variability (HRV) is reduced, we associate this reduction with an elevated risk for cardiovascular disease (Tsuji et al., 1994; Felber Dietrich et al., 2006), and an increase in mortality has also been reported in patients with circulatory system diseases (Ziegler et al., 2008; Drawz et al., 2013). HRV can be extracted from the ECG; we localize the R points and calculate the RR-intervals time series or tachograms. HRV is usually measured under well-controlled conditions over short periods of time of few minutes. Today, there is a greater use of ambulatory HRV measurement, usually with the use of a long-term ambulatory meter or Holter.

Long-term measurement makes it easy to assess the influence on HRV of activities of daily living as physical exercise. Changes in HRV induced by low or intense physical activity have been extensively reported (Tulppo et al., 1996; Aubert et al., 2003; Leicht et al., 2008; Goya-Esteban et al., 2012; Weippert et al., 2014; Taylor et al., 2017). In particular, in a seminal article, Karasik et al. (2002) noted how the dynamics of the heartbeat can change dramatically with physical activity. They noted that there are important differences in cardiac regulation associated with rest and exercise that cannot be clearly distinguished when analyzing the combined records of rest and exercise. They proposed that cardiac dynamics can be represented by a biased random walk toward some preferred levels of attraction: at rest, both the sympathetic and parasympathetic systems are active, and each attracts the walker to its own level. When the walker is between the two levels, each level biases in the opposite direction, virtually canceling the effect of the other. Therefore, the walker is free to move in both directions until he crosses either level and then is forced to return. With this proposal, they explained the crossover that they found in the scaling behavior of a higher value of the correlation exponent on short scales, where the fluctuations of the walker are not limited, to a lower value of the exponent on large time scales, where the walker’s dynamics is limited by the levels of attraction of the sympathetic and the parasympathetic.

During exercise, the sympathetic system dominates and the walker fluctuates around this level producing anti-correlated behavior on short time scales. However, since the level of attraction changes over time and as the walker follows these changes, fluctuations in the walk increase on intermediate time scales, causing a crossover to a more correlated behavior (Karasik et al., 2002).

Tachograms are non-linear time series, highly inhomogeneous, and non-stationary (Sugihara et al., 1996), they fluctuate in a complex way, suggesting that different parts of the signal have different scaling properties (Ivanov et al., 2001, 2002; Guzmán-Vargas et al., 2005; Voss et al., 2009; Shekatkar et al., 2017); therefore, non-linear methods can better capture changes in HRV that cannot be captured by linear methods. To analyze these time series, it has been used a lot of non-linear methodologies, for instance, detrended fluctuation analysis (Peng et al., 1995; Hu et al., 2001; Guzmán-Vargas et al., 2005; Muñoz Diosdado et al., 2005), time irreversibility (Porta et al., 2008; Visnovcova et al., 2014), fractal dimension (Higuchi, 1988; Eke et al., 2002), multifractal spectra (Goldberger et al., 2002; Aguilar-Molina et al., 2019), and a great number of other non-linear methodologies, and among them several entropy measures have been used to the same objective [see for instance (Shi et al., 2017) and references (Peng et al., 1995; Hu et al., 2001; Ivanov et al., 2001; Guzmán-Vargas et al., 2005; Muñoz Diosdado et al., 2005; Voss et al., 2009; Shekatkar et al., 2017) in that article]. The entropy of a dynamic system measures the information contained in its current state (Xiong et al., 2017), higher values of entropy indicate a more complex signal, and lower value of entropy implies less complexity of the signal. Entropy measurements can be applied to noisy processes with important stochastic components such as those that describe the dynamic activity of real-world systems, and they have been applied with great success to many fields of research, including HRV (Kurths et al., 1995; Porta et al., 1998; Vikman et al., 1999; Vigo et al., 2010; Voss et al., 2015; Xiong et al., 2017). The so-called conditional entropy includes a wide range of entropy measurements and estimates that have been recently proposed to quantify the complexity of a time series (Xiong et al., 2017). These measures, which include Approximate Entropy (ApEn; Pincus, 1991; Richman and Moorman, 2000), Sample Entropy (SampEn; Richman and Moorman, 2000; Lake and Moorman, 2011), Fuzzy Entropy (FuzzyEn; De Luca and Termini, 1972; Chen et al., 2007), corrected conditional entropy (Porta et al., 1998), and permutation entropy (Bandt and Pompe, 2002), are widely used for estimating conditional entropy in various fields.

One of the most important entropy measures is SampEn (Pincus, 1991; Richman and Moorman, 2000) which lately has been the most used because it has several advantages, one of which is that its values are stable with the size of the time series, SampEn was proposed because the first introduced kernel-based measure conditional entropy, the ApEn, was usually skewed (Xiong et al., 2017). One of the most recent and important articles on the calculation of conditional entropy is that of Xiong et al. (2017), that analyzed the dependence of the different entropies on the specific parameters of the estimator, as well as the effects of three types of non-stationarity due to the artifacts that are most commonly found in real data (trends, random peaks, and changes in local variance). In this article they presented for the first time a quantitative assessment of the impact on entropy measures of trends originating from the intrinsic dynamics of systems exhibiting multifractality properties. They considered the study of human heartbeat fluctuations in different physiological states and pathological conditions and their results evidenced advantages and pitfalls of entropy measures and estimators, as well as provided indications and recommendations for their optimal use in the study of real-world time series. In the HRV series analysis, when entropy measurements are applied correctly, they can characterize changes of specific types in the cardiac system that are associated with different physiological and clinical states. Xiong et al. established that a correct interpretation of the behavior of entropy measurements requires a clear understanding of the properties of the chosen specific measure and estimator, and an adequate choice of preprocessing applied to the measured signals. This is because when entropy methods are applied directly to the original HRV signals, there may be factors present in the data, such as long-range trends or correlations, which differently affect entropy measurements and estimators, and therefore can lead to inconsistent results and make interpretation difficult. In this work SampEn is used for the analysis of the RR-intervals time series, but the ApEn and Fuzzy En were also used to reinforce our results.

Exercise can bring out cardiovascular alterations that are not present at rest and can therefore be used as a means to assess cardiac function. Comparison between resting HRV time series or HRV time series during physical activity, using non-linear techniques has been an important topic (Ivanov et al., 1999b; Karasik et al., 2002) for several years, for example, in 1999 Ivanov et al. (1999b) compared the scaling properties of cardiac dynamics during sleep and wake periods for healthy individuals, subjects with congestive heart failure (CHF) and cosmonauts during orbital flight, and for the three groups, they found a higher degree of anti-correlation in the fluctuations in heartbeat during sleep compared to waking periods, and this difference from sleep-wake in the exponents of scale for all three groups is comparable to the difference between healthy and CHF patients. The observed differences in the scale that they reported (Ivanov et al., 1999b) are not simply explained by the different levels of activity. Karasik et al. (2002) studied the HRV of the heartbeat of healthy individuals at rest and during exercise. They focused on the correlation properties of the intervals formed by successive peaks in the time series and found significant scale differences between rest and exercise. For exercise, the interval series is anti-correlated on short time scales and correlated on intermediate time scales, while for rest they observed the opposite crossover pattern. As mentioned above, they suggested a physiological explanation to provide an intuitive explanation of the scale differences between rest and exercise. Of no less interest have been the analyzes of HRV observed due to circadian regulation and how it influences cardiac dynamics, for example, Ivanov (2007) hypothesized that, in addition to known periodic rhythms with a characteristic time scale, the mechanisms of sleep and circadian regulation can influence cardiac dynamics on a wide range of time scales and, therefore, could lead to systematic changes in the scale properties of heartbeat fluctuations. They found that scale-invariant characteristics of heartbeat dynamics, which have previously been associated with the underlying mechanisms of cardiac regulation, change significantly with the transition from sleep to wakefulness, through the stages of sleep and circadian phases, both in healthy and pathological conditions.

The endogenous circadian pacemaker is known to influence physiological functions, it is normally synchronized with the sleep-wake cycle, and HRV often exhibits complex continuous fluctuations, even in healthy resting conditions (Ivanov et al., 1996, 1999a,b; Bunde et al., 2000; Ivanov et al., 2001; Hu et al., 2004). Heartbeat fluctuations in healthy subjects possess a self-similar temporal structure related to the underlying cardiac control mechanism, characterized by long-range correlations over a wide range of scales (Peng et al., 1995; Ivanov et al., 2001). These characteristics change with sleep-wake states (Ivanov et al., 1999b; Bunde et al., 2000; Kantelhardt et al., 2002; Penzel et al., 2003; Hu et al., 2004), exercise (Karasik et al., 2002; Martinis et al., 2004), and in pathological conditions (Goldberger, 1996; Ho et al., 1997; Ivanov et al., 2001). Ivanov et al. (2007) established in a relevant article that physical activity affects the average heart rate, but it is not known how the dynamic scale-invariant measures of these two physiological variables are related. They investigated the activity and heartbeat data in healthy subjects at all circadian phases and determined whether circadian influences on static or dynamic characteristics of heart rate regulation are decoupled from circadian influences on activity regulation. They noted that exercise, may also be an independent contributing factor to increased cardiac risk when living outside of the laboratory setting.

The stress test (ST) is a procedure diagnostic that assesses the response of the heart to a progressive physical exercise. The ST is one of the most common non-invasive tests in cardiology to establish or confirm the diagnosis and prognosis of heart disease and to assess the effect of its treatment. The presence of cardiovascular abnormalities can be manifested by alterations of the parameters that are determined during the test. Thus, undergoing additional work for the heart, while watching the patient and his ECG monitors, it is possible to discover heart problems that are not evident in the subject at rest. It has been shown that heart rate increases during exercise due to both parasympathetic withdrawal and increased sympathetic activity. The relative role of these two impulses depends on the intensity of the exercise (Aubert et al., 2003). It has been reported in animal models that obtaining physical conditioning, before an induced pathology, can reduce the problems caused by the disease (Amaral et al., 2016). In this work we measured how active the study subjects were by using the IPAQ (International Physical Activity Questionnaire; Booth, 2000; Booth et al., 2000; Craig et al., 2003; International Physical Activity Questionnaire [IPAQ], 2016) questionnaire, although there are different ways to study physical activity data, the use of this scoring method enhances the comparability between surveys. IPAQ is an instrument specially designed to monitor the population of physical activity among adults.

In this work the SampEn, ApEn, and FuzzyEn algorithms were applied to the analysis of tachograms obtained from healthy adults both at rest and during STs. The ST consisted of walking on a treadmill for 30 min. We worked with three groups of people: A group of young sedentary people around 20 years of age, another group of adults around 50 years also of sedentary habits, and another group of people that without being athletes perform physical activity daily on a regular basis. Significant differences were found between the entropy values from the rest series and entropy values during the STs for the three groups. In the sedentary groups (both young and middle-aged adults) there is a decrease in the entropy. But in the case of people who regularly do exercise there is a different trend. Finally, in a test of longer duration (60 min) entropy decreases as the ST progresses, this last test was performed both on treadmill and running track and in both cases the trend is the same. Insights about the influence of physical condition on the entropy values can be obtained from the results.

The paper is organized as follows. The method and the procedure to obtain the data are described in section “Materials and methods.” In section “Results” we present our results and in the Discussion section we analyze and interpret the results obtained for the entropy in the different situations. Finally, we present our conclusions.

## Materials and Methods

From a time series with *N* points given by the expression {*x*_*i*_, 1 ≤ *i* ≤ *N*}, a set of vectors of length *m* is constructed. There are various methods to evaluate entropy (Kurths et al., 1995), we have chosen three of them in the present work, that we will describe below. SampEn is our principal method and ApEn and FuzzyEn have been used for comparison purposes and to reinforce our results. SampEn does not present major changes due to the length of the time series. In previous research it had been proven that the method gives excellent results for synthetic and physiological time series (Muñoz-Diosdado et al., 2017).

### Algorithm of Approximate Entropy

The evaluation of entropy can be thought of as the conditional probability that two templates matching within an arbitrary tolerance will continue to match at the next point. Entropy is estimated with greater precision when more events are counted. For a length *m < N* and starting point *i*, the template *x*_*m*_(*i*) is the vector containing the *m* consecutive intervals *x*_*i*_, *x*_*i*+1_, ⋯, *x*_*i*+*m*−1_ (Richman and Moorman, 2000), *m* is the length of sequences to be compared, and *r* is the tolerance for accepting matches. The ApEn measures the irregularity of a time series by comparing subseries of length *m*, each subseries represents a pattern, which is subsequently compared with the other patterns of the same length. Therefore, the more repeatability there is between the patterns, the more predictable, or regular the time series will be. For a time series {*x*_*i*_,  1 ≤ *i* ≤ *N*} ApEn can be calculated by the following procedure (Shi et al., 2017).

$${\vec{x}}_{i}=\left(x_{i},x_{i+\tau },\cdots ,x_{i+\left(m-1\right)\,\tau }\right)$$

Here *m* indicates the embedding dimension and τ the time delay. In this way, *N*−*m*τ +  1 vectors of length *m* can be constructed, for example, for τ = 1 and *m* = 3, the first three vectors are: (x1,x2,x3),x→2=(x2,x3,x 4)⁢y⁢x→3=(x3,x4,x5).

The distance between the vectors ${\vec{x}}_{i}$ and ${\vec{x}}_{j}$ is defined as the maximum difference in absolute value of the components of the vectors, that is,

$$d_{i,j}=max\left(|{\vec{x}}_{i+k}-{\vec{x}}_{j+k}|,0\leq k\leq m-1\right).$$

For a vector ${\vec{x}}_{i}$ of length *m* we calculate the percentage of vectors ${\vec{x}}_{j}$ whose distance is less than a threshold factor *r*, that is, *d*_*i*,*j*_ ≤ *r*:

$$C_{i}^{m}=\frac{N_{i}^{m}\,\left(r\right)}{N-m}$$

Where $N_{i}^{m}\,\left(r\right)$ is the total number of vectors v “similar” to the vector ${\vec{x}}_{i}$, on the other hand. Is important to consider that if the series are not normalizad the *r* factor is normally considered as a fraction of the standard deviation of the time series, (σ_*x*_ = *std*(*x*_*i*_)), usually *r* =  0.2σ_*x*_ (Richman and Moorman, 2000). In the evaluation of entropy it is quite common that the series is normalized with a standard deviation equal to 1, that is why the tolerance in that case is simply equal to *r*.

The average of the percentages for the time series {*x*_*i*_, 1 ≤ *i* ≤ *N*} is defined as*:*

$$\Phi^{m}\,\left(r\right)=\sum_{i=1}^{N-m}\frac{\ln\left[C_{i}^{m}\,\left(r\right)\right]}{N-m}$$

The above process is repeated to calculate Φ^*m*+1^ (*r*). ApEn of the time series is calculated by:

$$A\,p\,E\,n\,\left(m,r\right)=\Phi^{m}\,\left(r\right)-\Phi^{m+1}\,\left(r\right)$$

### Algorithm of Sample Entropy

The sampling entropy (SampEn) was developed because it has a better representation of the entropy of the analyzed signals compared to the ApEn (Richman and Moorman, 2000). The motivation for this method is the classification of the complex system that includes both deterministic and stochastic characteristics of time series with a limited number of data points compared to other measures such as the correlation dimension (Grasberger and Procaccia, 1983).

In a similar way to what is done for the ApEn, the vectors ${\vec{x}}_{i}$ and ${\vec{x}}_{j}$ of length *m* are obtained.

Thus, in this context two vectors ${\vec{x}}_{i}$ and ${\vec{x}}_{j}$ are similar if they meet that *d*_*i*,*j*_ < *r*, where *r* is a threshold value that depends on established conditions. Analogously to the case of ApEn it is chosen as: *r* =  0.2σ_*x*_ if the time series is not normalized.

For a length *m* and threshold *r*, the number of vectors of length m similar to ${\vec{x}}_{i}$ is calculated by:

$$n_{i}^{m}=\sum_{\begin{array}{c} j=1\\ j\neq i \end{array}}^{N-m}\delta \,\left(\text{i},\text{j},\text{m},\text{r}\right)$$

Where

$$\delta \,\left(\text{i},\text{j},\text{m},\text{r}\right)=\left\{ \begin{array}{c} 1\;i\,f\;d_{i,j}<r\\ 0\;o\,t\,h\,e\,r\,w\,i\,s\,e \end{array}\right.$$

It is noteworthy that *i*≠*j*, means that the self-comparison of a vector with itself is not considered in the sum. The similarity $A_{i}^{m}$ of the vector set ${\vec{x}}_{i}$ y ${\vec{x}}_{j}$ for a length *m* is calculated by:

$$A_{i}^{m}=\frac{1}{N-m}\,n_{i}^{m},i=1,2,\cdots ,\text{N-m}$$

Now the average similarity can be obtained by the expression

$$A^{m}=\frac{1}{N-m}\,\sum_{i=1}^{N-m}A_{i}^{m}$$

Using the same time series and tolerance *r*, the average similarity is calculated for a vector of length *m+1*, that is, it is calculated *A*^*m* + 1^.

The so-called SampEn is obtained by,

$$S\,a\,m\,p\,l\,e\,E\,n\,\left(m,r\right)=-\text{ln}\,\frac{A^{m+1}}{A^{m}}$$

### Algorithm of Fuzzy Entropy

The FuzzyEn is a methodology very similar to the SampEn, as in the previous cases the vectors of longitude *m*, ${\vec{x}}_{i}$ and ${\vec{x}}_{j}$ are defined from the time series {*x*_*i*_, 1 ≤ *i* ≤ *N*}. The counting of similar vectors is now done by changing the reference *r* by the membership function:

$$\mu \,\left(d_{i,j},r\right)=e\,x\,p\,\left(-l\,n\,\left(2\right)\,{\left(\frac{d_{i,j}}{r}\right)}^{2}\right)$$

Where *d*_*i,j*_ represents the distance:

$$d_{i,j}=\max\left(\left|\left[{\vec{x}}_{i+k}-\bar{x_{i}}\right]-\left[{\vec{x}}_{j+k}-\bar{x_{j}}\right]\right|,0\leq k\leq m-1\right).$$

Where

$$\bar{x_{i}}=\frac{1}{m}\,\sum_{k=0}^{m-1}{\vec{x}}_{i+k}$$

In this case, $\bar{x_{i}}$ and $\bar{x_{j}}$ are, respectively, the averages of the vectors ${\vec{x}}_{i}$ and ${\vec{x}}_{j}$, this eliminates the local average of vectors. In this way, FuzzyEn evaluates the similarity between vectors based primarily on their shape. From the above it is obtained that the degree of similarity is in this case:

$$B_{i}^{m}=\frac{1}{N-m}\,\sum_{\begin{array}{c} j=1\\ j\neq i \end{array}}^{N-m}\mu \,\left(d_{i,j},r\right),i=1,2,\cdots ,\text{N-m}$$

From now on the calculations are identical to those made for the SampEn. Again *r* =  0.2σ_*x*_ as in the previous methods. The average similarity for the FuzzyEn is:

$$B^{m}=\frac{1}{N-m}\,\sum_{i=1}^{N-m}B_{i}^{m}$$

With the same parameters *B*^*m* + 1^ is evaluated, finally FuzzyEn is obtained:

$$f\,u\,z\,z\,y\,E\,n\,\left(m,r\right)=-\text{ln}\,\frac{B^{m+1}}{B^{m}}$$

All three methods require values of the parameters *m*, *r*, and *τ*, based on our previous experience and that reported by other authors (Pincus, 1991; Richman and Moorman, 2000; Lake and Moorman, 2011; Muñoz-Diosdado et al., 2017; Shi et al., 2017) we obtained better results for the set of values: of *r* = 0.2, *m* = 2, and *τ* = 1 for present calculations, although we had previously used *m* = 3 and the results were also good. For example, Shi et al. (2017) used the values of *m* = 2, *r* = 0.1, and *τ* = 1 for the calculations they made, for ApEn, SampEn, and FuzzyEn and other entropies they used. Richman and Moorman (Richman and Moorman, 2000) showed that for series with more than 100 points, the SampEn values with *m* = 2, and *r* = 0.2 have deviations less than 3% of the predicted values, while for very short series, the values obtained for SampEn have deviations up to 35%. Lake and Moorman (2011) used *r* = 0.2 and different values of *m*, they obtained excellent results for both *m* = 1 and *m* = 2. Muñoz-Diosdado et al. (2017) also obtained good results using *r* = 0.2 and *m* = 3, this *m*-value was chosen because they analyzed series with 10,000 points. Pincus (1991) analyzed the ApEn for values of *m* = 1, 2, and *r* between 0.1 and 0.25. We can show, for time series generated by us for white, Brownian and pink (1/f) noises, the performance of these three methodologies for different parameter values. The self-affine series were generated with *N* = 3000 data and different values of the spectral exponent β: β = 0 (white noise), β = 1 (pink noise), and β = 2 (Brownian noise), to generate the series we followed the methodology of the references (Mandelbrot and Van Ness, 1968; Malamud and Turcotte, 1999; Gálvez Coyt et al., 2010).

**FIGURE 1:**
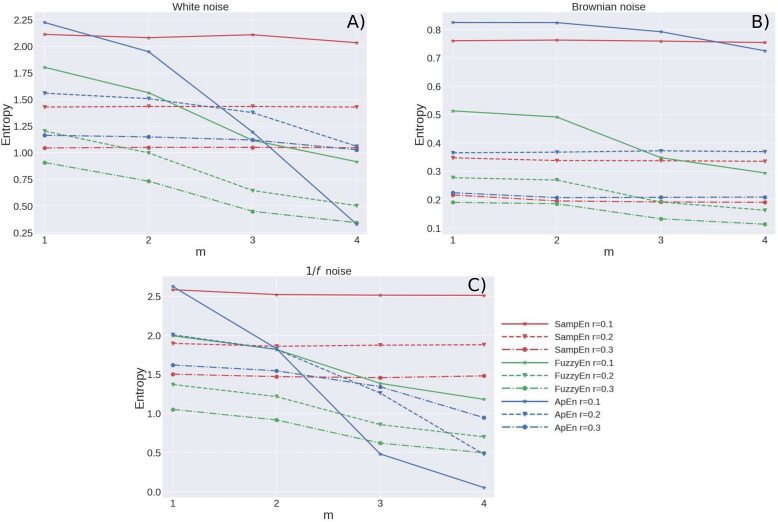
Values of SampEn (**A**; red), ApEn (**B**; blue), and FuzzyEn (**C**; Green) for self-affine time series with 3000 data and different values of *m* (1, 2, 3, and 4) and *r* (0.1, 0.2, and 0.3). SampEn behaves stably for different values of *m*, the variations in entropy values with respect to *r* are normal, the largest entropy values correspond to the smallest values of *r* and vice versa, the smallest entropy values correspond to the larger values of *r*.

As a comparison of the three methods to calculate the entropies, three time series of length *N* = 3000 data were used, the first series corresponds to white noise, the second to Brownian noise and the third to 1/f noise. For the three series, the three entropies were calculated for values of *m* = 1, 2, 3, and 4 and *r* = 0.1, 0.2, and 0.3 (multiplied by the standard deviation of the time series). All the results obtained are summarized in Figure 1. Figure 1A shows the results for white noise, Figure 1B for Brownian noise and Figure 1C for 1/f noise. Since they are not real-world series, they were not preprocessed. As can be seen, the highest entropy values are obtained for 1/f noise, then for white noise and finally for Brownian noise. Considering the variation of the entropies with the *m*-value, it turns out that the graphs for the SampEn (in red color) are practically horizontal lines for all cases (*m* = 1, 2, 3, and 4). For the ApEn (blue), the variations are stronger in all cases, although in the case of Brownian noise, their variations are not as dramatic as in the case of 1/f noise and white noise. The FuzzyEn (green) also presents important variations, but for cases *m* = 3 and 4, its values for *m* = 1 and *m* = 2 are very similar, so its performance is better for *m* = 1 and 2. This last observation is also valid for the ApEn. In other words, the SampleEn is the most stable when changing the size of the *m*-value. As for the variation of the entropies with respect to *r*, it causes a vertical displacement for all of them. This is a well-known fact: For instance, Xiong et al. (2017) have reported that the width of *r* determines the size of the cells used for the probability estimation: when it decreases, less *r* points are included in the cell used to estimate the probabilities; therefore the estimated probabilities are lower, giving higher entropy estimates. On the contrary, when *r* increases, more points are included in the vicinity of any reference point, which increases the estimated probability and therefore leads to lower entropy estimates. Since taking the tolerance equal to 0.2σ_*x*_ gives the intermediate values for all entropies, in this work we chose to evaluate the entropies with this tolerance value.

In this manuscript we work with HRV series at rest and HRV series when exercising, we report the observed changes in entropy, that is, we evaluate SampEn, ApEn, and FuzzyEn and we calculate the exercise-rest variations and report such changes, obviously the changes do not have the same values, but the trends are the same, that is, if we notice that there is a decrease in entropy, such decrease is observed in the three entropies, although the average changes do not have the same value.

Therefore, although the three entropies were evaluated, the values shown in the graphs correspond to SampEn if not stated otherwise, because as shown in Figure 1, this entropy has more stable values and the calculations are more robust than the other entropies, the choice of *m* = 2 and *r* = 0.2 provides good estimates for the three entropies, but mainly for SampEn.

We know that the accuracy of the estimates is highly dependent on the time series length, so very long time series would be needed to yield accurate estimation of conditional entropy if there are strong positive long-range correlations. However, based on our experience and in the other authors (Richman and Moorman, 2000; Lake and Moorman, 2011; Muñoz-Diosdado et al., 2017; Shi et al., 2017), SampEn is the most stable entropy with respect to the time series length. In our case, we chose to use series of the same length to assess entropy.

### Physionet

**FIGURE 2:**
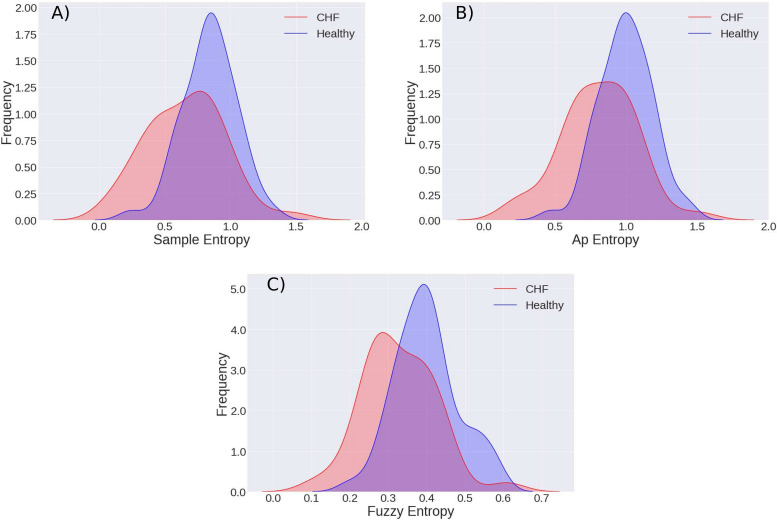
Entropy comparison of the asleep period time series of the 39 congestive patients and 52 healthy subjects. The time series correspond to the 6-h records while the subjects are asleep. **(A)** Sample Entropy. **(B)** Approximate Entropy. **(C)** Fuzzy Entropy.

To illustrate how the methods work and how they can give good results, SampEn, ApEn, and FuzzyEn were implemented in the analysis of tachograms obtained from the beat to beat time of the Physionet datasets of CHF RR Interval Database (Goldberger et al., 2000) with 29 CHF patients, the BIDMC CHF Database with 15 CHF patients subjects, and the Normal Sinus Rhythm RR Interval Database with 54 patients with normal sinus rhythm. We obtained 6-h subseries when the subjects were sleeping, but it was only possible to obtain these subseries for 52 healthy subjects and 39 patients. The results are shown in Figure 2, we applied T-Student tests with a significance level of 0.05 to show that there is a statistically significant difference between the average entropies for healthy subjects and CHF patients, the same behavior is observed for SampEn, ApEn, and FuzzyEn. For SampEn, we obtained for the patients with CHF: ${\bar{S\,a\,m\,p\,E\,n}}_{C\,H\,F}=0.6567\pm 0.2923$ and for the healthy ${\bar{S\,a\,m\,p\,E\,n}}_{H}=0.8398\pm 0.2039$, the values are statistically different with a *p*-value of *p*_*SampEn*_ = 0.00060; for ApEn we obtained: ${\bar{A\,p\,E\,n}}_{C\,H\,F}=0.8067\pm 0.2602$ and ${\bar{A\,p\,E\,n}}_{H}=0.9967\pm 0.1851$ and these average values are statistically different, *p*_*ApEn*_ = 0.0001, and finally, for the FuzzyEn: ${\bar{F\,u\,z\,z\,y\,E\,n}}_{C\,H\,F}=0.3273\pm 0.0938$ and ${\bar{F\,u\,z\,z\,y\,E\,n}}_{H}=0.3995\pm 0.0823$, and there is also a significant difference, with *p*_*FuzzyEn*_ = 0.0002. In general, there is a diminution in the entropy associated to the CHF disease (Martinis et al., 2004; Goya-Esteban et al., 2012). After appreciating that the three entropies could properly differentiate between healthy and congestive subjects, the methods were used for people in a cardiac stress condition, as we will describe in the following. But in order not to be repetitive and since the same behavior is observed for the three entropies, the graphs illustrate preferably the SampEn.

### Physical Activity

Physical inactivity is a global health problem, one of the most standardized approaches to measure it was proposed by Craig et al. (2003), based on a proposal by Booth (2000). Its objective was to develop an appropriate measure to assess the levels of physical activity of the population in all countries.

The questionnaire they proposed, the IPAQ, was designed to be used in adults between 18 and 65 years old. There is a short version that provides information on the time spent walking, in activities of vigorous and moderate intensity and in sedentary activities. The long version, which was the one we use in this work, was designed to collect detailed information within the domains of domestic and garden work activities, occupational activity, transportation and physical activity in leisure time, as well as sedentary activity. IPAQ instruments have been used to collect reliable and valid physical activity data in many countries (Booth et al., 2000; International Physical Activity Questionnaire [IPAQ], 2016).

The detailed explanation of the method can be consulted in International Physical Activity Questionnaire [IPAQ] (2016); here we will only say that the questionnaire allows classifying populations into three levels of physical activity: high, moderate, and low.

High: This category was developed to describe the highest levels of physical activity. Although the greatest health benefits are associated with higher levels of activity, there is no consensus on the exact amount of activity to obtain maximum benefit. The IPAQ Research Committee proposed a measure that is equivalent to at least 1 h per day or more, of physical activity above the baseline level of physical activity (walking about 5000 steps per day), this category is considered for those who walk at least 12,500 steps per day, or the equivalent in moderate and vigorous activities. This represents at least one more hour of activity of moderate intensity above the baseline level of activity, or half an hour of activity of vigorous intensity above baseline levels daily.

Moderate: This category corresponds to an activity level equivalent to half an hour of physical activity at least of moderate intensity on most days.

Low: This is simply defined as not meeting any of the criteria for any of the other classifications.

In this paper, we want to highlight the differences in the calculated entropy values for sedentary people and for active people, comparing the entropy values at rest and exercising. Therefore, we define a person as sedentary if their physical activity result using the IPAQ questionnaire is “low”; a person is active if they obtained a result of “high” in the physical activity evaluation according to the IPAQ questionnaire. To avoid confusion, the individuals who obtained “moderate” in the evaluation were excluded from the present study.

### Stress Test

Tachograms of thirty-eight subjects were analyzed at rest and in a cardiac ST. The population was divided by age and by the amount of physical activity according to the IPAQ classification. Twenty-five young sedentary subjects were analyzed at rest and in a cardiac ST. The subjects were 5 men and 20 women with an average age of 23 years old. Another 6 young subjects who regularly do exercise every day and that are in good physical condition even though they are not athletes, with an average age of 23 years were also considered for comparison purposes.

The measures were repeated for six middle-aged subjects with an average age of 50 years, two women and four men, all of them are sedentary. We also have two active middle-aged adults. The conditions were the same that for the first group, but we decreased the speed of the ST to 3.5 miles per hour. The time series used to perform the calculations are available in the Supplementary Material of this article. Complete ECG records are available on request to the correspondence author.

For each subject, the personal information was collected, including age and gender. Subjects with any disease that could affect the cardiovascular system were not included in the study. Subjects should not be taking medications previous and during the STs. They were asked to have adequate sleep during the previous night. All tests were performed in the morning (∼10 a.m.) in a quiet room with a temperature between 20 and 21 centigrade degrees.

The body mass indices of the subjects are as follows, for active youth it is 24.01 ± 1.76, for sedentary youth it is 24.00 ± 4.07, and for sedentary adults it is 26.11 ± 2.69.

**FIGURE 3:**
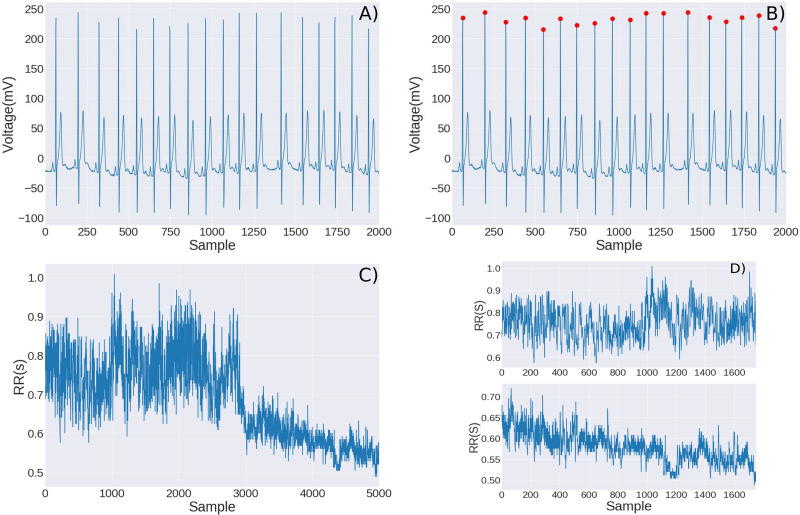
We show ECG and RR time series for a sedentary young subject. First, a segment of the digitized ECG signal obtained with the Holter **(A)**, the localization of the R points was obtained with an algorithm previously designed **(B)**, the difference (in seconds) between the R points gives the RR time series or tachogram, note that it is easily distinguishable when the subject is at rest and when the subject is walking in the treadmill **(C)**. The tachogram belongs to a healthy young subject, the first part belongs to the rest period, and the second half of the time-series belongs to the cardiac stress test, we can obtain subseries corresponding to the rest and to the exercise **(D)**. Note that there is a decreasing trend in RR intervals as time progresses and the individual experiences some fatigue.

For each recording, there were two measures, a 60 min’ rest record and a 30 min’ cardiac ST was recorded. For this experimentation, the study was designed by taking the patients into a complete rest state while ECG recording was obtained with a Fukuda Denshi Holter monitor model FM-150 with sampling frequency of 125 Hz, once the 60 min’ rest period had finished, the subjects were taken into a commercial electric treadmill at 4.0 miles per hour (mph) during 30 min. The beat to beat signal or tachogram was obtained for each digitalized ECG and then processed with the entropy algorithms. One of the measured time series is shown in Figure 3, in the tachograms it is easily recognizable when the subjects are at rest and when they are doing the walking test. Therefore, it was easy to obtain the tachograms at rest and the tachograms at the ST. It was mentioned in the introduction section that Karasik et al. (2002) provided a qualitative explanation of the remarkable differences in the amplitude of fluctuations at rest and during exercise, when the walker is between the two levels of attraction, no net force acts on him, so there is a small probability of going several steps in the same direction and in the case of a single level of attraction, there is a restriction that changes its direction. Therefore at rest when both levels are active, the fluctuations are greater compared to exercise when there is a single level of attraction (see Figure 3C). As the HRV time series are complex, physiological interpretations based on entropy measures should be provided with caution (Xiong et al., 2017; Faes et al., 2019). As Xiong et al. (2017) have stated, entropy measurements can only differentiate changes of specific types in cardiac dynamics and that proper preprocessing is vital for correct estimation and interpretation. They recommended some strategies to analyze the HRV time series. The entropy measurements depend on the length of the time series, but as we mentioned earlier, SampEn is very stable with respect to the length of the time series, and we take the length of the series of the same length, at rest we analyze series with 1024 points and in the ST we also analyzed 1024 points. Although trends have a big impact on the detection of the dynamical complexity of stochastic processes, we decided not to eliminate the trends in our data because for two main reasons, the time series are quite short and because we want to analyze the effect that fatigue has on the time series. Spikes are commonly encountered in a large variety of measurements; we took special care to eliminate the artifacts. Fortunately, since the measurements were made at rest and at low speeds, the operation of the Holter Fukuda Denshi FM 150 is very stable and the measured signal was very clean, there were few spikes, their values were replaced by the average of the previous and next data. Although the data were normalized for the calculation of entropy, the calculations were also made without normalization and the same results were obtained. As we can see in our time series (available in Supplementary Material) we did not have segments of high variance, and fortunately the presence of this sort of non-stationarity does not affect results as much as other types of non-stationarity (Xiong et al., 2017).

An important point to highlight is the fact that the time series for the STs were not taken since the subject got on the treadmill, it took a few seconds for the signal to stabilize, that is, we did not take points in the rest-exercise transition region.

Long-lasting one-h records were also measured for seven sedentary youth who walked at a speed of 3.5 miles per hour. These records were 2-h length, 1 h for the rest period, and 1 h for the cardiac stress episode. The tachogram that corresponds to the ST was divided into four parts, equivalent of 15 min of recording and for each 15-min segment we calculated the entropies.

This research was approved by the Secretaría de Investigación y Posgrado of the Instituto Politécnico Nacional of Mexico with the grants SIP20171974, SIP20182121, and SIP20196318 and by the Secretaría de Educación, Ciencia y Tecnología (SECTEI) of Mexico City with the project SECTEI/271/2019. Given that the project only considers measuring the ECG in healthy people at rest and walking slightly at low speeds, we were not required to present the proposal to the Ethics Committee of the Institution.

The participants were voluntary and signed the informed consent form. Medical staff confirmed that the participants were healthy, individuals who presented cardiovascular diseases or risk factors such as hypertriglyceridemia, hypertension, or diabetes were excluded. Specialized personnel were always present during the measurements. All the research was in compliance with the Ethics Code of the World Medical Association (Helsinki Declaration).

## Results

**FIGURE 4:**
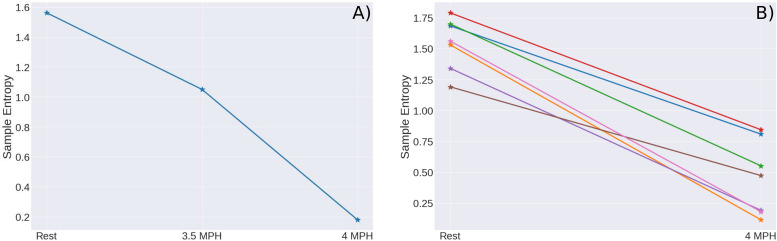
**(A)** SampEn for one subject where the first record belongs to the rest period, the second record to a cardiac stress condition at 3.5 mph and the third measure to a cardiac stress condition at 4 mph. **(B)** SampEn values for rest and cardiac stress tests of one subject in seven measures. The mean of SampEn at rest is 1.3541 ± 0.187 and at cardiac stress SampEn mean is 0.4344 ± 0. 2475.

A general behavior is observed for all sedentary people; at rest they have a higher entropy value than when they are exercising. This difference in entropy is statistically significant. This behavior is shown in Figure 4A for a sedentary young person, the first point corresponds to the value of SampEn at rest, the second point to the value obtained when he walked on the treadmill at a speed of 3.5 mph, then he was asked to rest for half an hour and to walk another 30 min at a speed of 4 mph, the third point corresponds to the value of the SampEn for the speed of 4 mph. SampEn decreases as the speed increases. As the same behavior is observed for ApEn and FuzzyEn we do not show the corresponding figures.

The behavior of entropy is the same for all sedentary, similar to that of Figure 4A, we mean that in all the time series of sedentary subjects, the 25 young subjects and the 6 middle-aged adults, this decrease in entropy is observed. So SampEn (and ApEn and FuzzyEn) decreases while stressing the hearth of the sedentary subjects. In order to prove that the obtained result was not random and thinking whether or not the results are reproducible, one sedentary subject was tested seven times in order to confirm that the observed pattern of the graph was reproducible. In Figure 4B we can observe the comparison between seven different experiments with the same subject. As we can observe, in all the measures the pattern can be observed. There are variations, as expected in these complex systems, but in general the pattern is repeated. As observed in the Figure 4A, the loss of entropy when stressing the hearth of the subjects is notorious, this can be seen as a negative slope while drawing a line between both values as we can visualize in Figure 4B.

**FIGURE 5:**
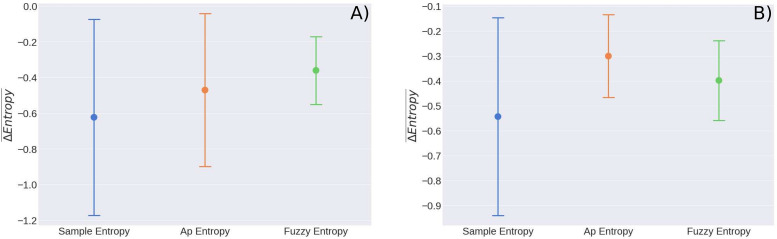
SampEn values for rest and cardiac stress tests for **(A)** 25 young subjects and **(B)** six middle aged adults. The speeds for the stress tests were 4 mph and 3.5 mph, respectively. There are no significant statistical differences.

We show in Figure 5A, the values of slope obtained for the 25 sedentary young person’s when using the three different entropy methods mentioned before, and with the aim of comparison we show in Figure 5B, the results obtained for the six middle-aged adults with low physical activity. As mentioned, all entropy decreases for sedentary subjects when doing the ST, but as can be seen in Figure 5, the difference between youth and adults is not significant. The average change in Entropy for young persons is $\bar{\Delta \,S\,a\,m\,p\,E\,n}=-0.62\pm 0.56$, $\bar{\Delta \,A\,p\,E\,n}=-0.47\pm 0.43$, and $\bar{\Delta \,F\,u\,z\,z\,y\,E\,n}=-0.36\pm 0.19$ and the values for adults with a sedentary lifestyle are $\bar{\Delta \,S\,a\,m\,p\,E\,n}=-0.54\pm 0.43$, $\bar{\Delta \,A\,p\,E\,n}=-0.3969\pm 0.3680$, and $\bar{\Delta \,F\,u\,z\,z\,y\,E\,n}=-0.39\pm 0.17$.

**FIGURE 6:**
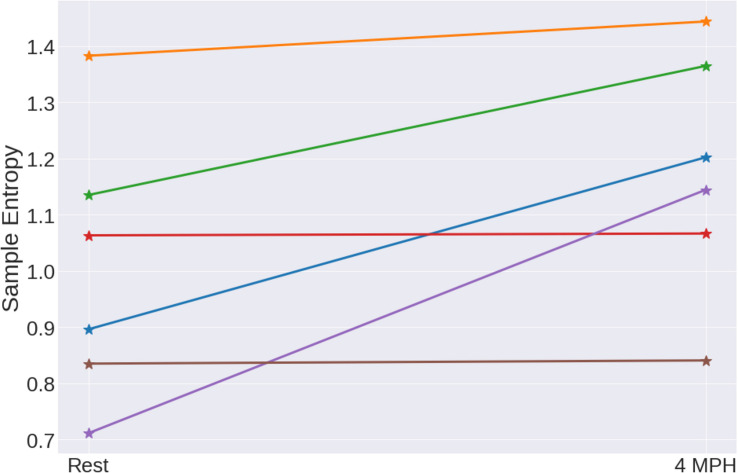
Sample entropy values for rest and cardiac stress tests for six young subjects that do regular physical activity.

The other group of six young subjects that regularly do exercise was analyzed with the same methodology. These six evaluated individuals have a particular difference with respect to the other young subjects, probably due to physical activity. In comparison with the observed results in Figure 5, the straight lines have in average positive values, that is, the entropy of the time-series tends to increase or maintain its values while performing the cardiac stress episodes (Figure 6). But it should be clear that this increase in entropy is observed when going from rest to the ST when walking at 4 miles per hour, if we increase the speed, eventually the entropy decreases (see Discussion section).

Entropy values behave similarly for people that have similar patterns of physical activity independently of their age. In order to stress this idea, a comparison of both populations was made. Adults and young people with low physical activity were grouped together and were compared with adults and young people with high levels of physical activity.

**FIGURE 7:**
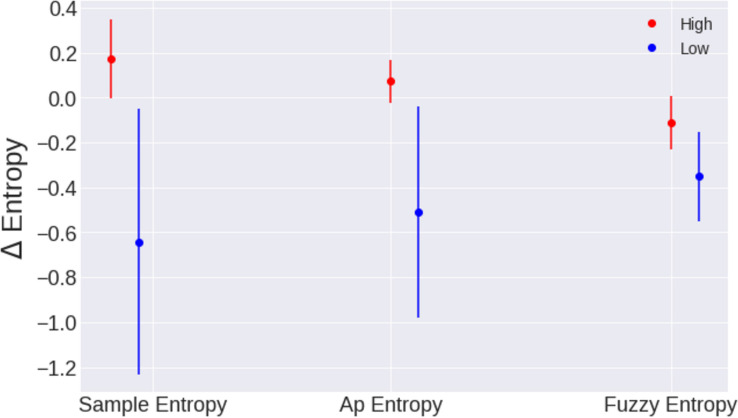
Comparison of people with high physical activity and people with low physical activity for the three entropy methods, ApEn, FuzzyEn, and SampEn. The means of these populations are statistically different with *p* values 0.0011, 0.0052, and 0.0033 for the three methods, respectively.

For this test, the slope of the line between the value of entropy at rest and the ST was compared for the two groups for the three entropy methods having the following values for the group of sedentary people are $\bar{\Delta \,S\,a\,m\,p\,E\,n}=-0.64\pm 0.69$, $\bar{\Delta \,A\,p\,E\,n}=-0.51\pm 0.47$, and $\bar{\Delta \,F\,u\,z\,z\,y\,E\,n}=-0.35\pm 0.20$. The following values for people who reported to have physical activity in a daily basis: $\bar{\Delta \,S\,a\,m\,p\,E\,n}=0.17\pm 0.07$, $\bar{\Delta \,A\,p\,E\,n}=0.07\pm 0.09$, and $\bar{\Delta \,F\,u\,z\,z\,y\,E\,n}=-0.11\pm 0.12$. This comparison was made for the three types of entropy as it is possible to visualize in Figure 7. As always, the sampling entropy is the one that gives us the best results.

**FIGURE 8:**
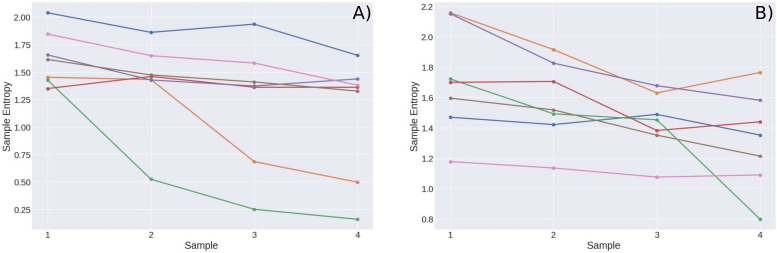
Results of sample entropy for 1-h cardiac stress tests in **(A)** a treadmill at 3.5 mph and **(B)** a track at approximately 3.5 mph.

In the last experiment, we are interested in seeing how entropy varies as the test progresses, that is, as fatigue accumulates. As previously mentioned, seven sedentary young subjects were asked to walk during an hour after a rest period of an hour. The entropy was calculated every 15 min, having a total of four SampEn results for each subject. These results are shown in Figure 8A. The speed of the treadmill was 3.5 mph. The graph in Figure 8B, belongs to the same subjects, but this time they did not walk on the treadmill but on a running track, the speed is approximately of 3.5 mph although it is difficult to control the speed in the running track.

## Discussion

The present work shows that there is a variation in SampEn, ApEn, and FuzzyEn values for beat to beat time-series of subjects in different conditions. It has been reported that the tachograms of healthy persons tend to have higher entropy values than the tachograms of the population diagnosed with CHF, this lack of entropy implies a loss of complexity of the signal. This is similar to what we observed in the present work, when sedentary people are at rest their tachograms have SampEn, ApEn, and FuzzyEn values that decrease when they undergo STs, therefore the beat to beat time series are less complex when they are doing exercise.

In order to secure that the results were trustworthy and the observed pattern where entropy decrease in cardiac stress situations was replicable, the measures were developed in different days for a single patient reproducing the conditions of the test. The analysis of this experiment allowed us to affirm that the results are reproducible (Figure 4B). As known, cardiac system has complex dynamics and there are many factors that affect the cardiac rhythm, in accordance with this, the measures observed are not identical, but the tendency of entropy to decrease during the ST is always observed.

While comparing the subjects, a difference between those who develop regular physical activity and the ones who do not was observed. In contrast with the common tendency showed by most of the tested subjects, a slight increase of the average entropy values was observed in subjects with regular exercise on a daily basis. In contrast with the subjects who live more sedentarily, the entropy measurement of the time series of the cardiac ST may have an increase compared to the resting time series, or it tends to maintain its values, or, in the event of a decrease in entropy, this decrease is small.

Looking for a difference between young sedentary subjects and middle age sedentary adults, the pattern of decreasing entropy was appreciated for both groups (Figure 5). But there are not significant but there are no statistically significant differences between both groups.

In the one-h cardiac STs, the tendency observed in the previous experiments is more evident. Comparing the different values of entropy at different times of the test, it tends to decrease while the time goes on. The cardiac stress not only depends on the speed of the test, but in the duration of it.

**FIGURE 9:**
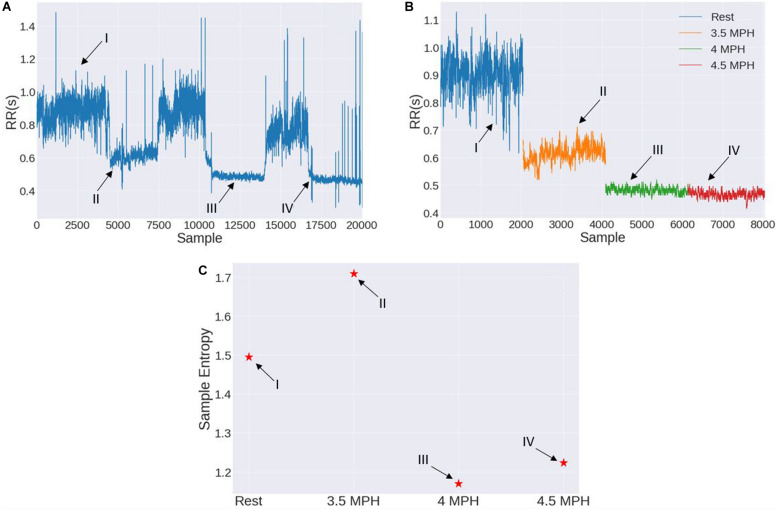
This figure shows a stress test performed by an active person. **(A)** Original time series, **(B)** the stages of the stress test, and **(C)** sample entropy for the time subseries of each stage. The different stages of the test are: **(I)** 1 h at rest, **(II)** walked half an hour at a speed of 3.5 mph, **(III)** walked at 4.0 mph preceded by a half-hour break, **(IV)** walked at 4.5 mph, also preceded by a half-hour break. In this figure we can see what has already been discussed in the article, an active person has an entropy-value at rest **(I)**. When walking at moderate speed the value of entropy tends to increase or maintain **(II)**, which is exactly the opposite of what happens with sedentary people. It is necessary to increase the walking speed so that the decrease in entropy is noticed **(III,IV)**.

Summarizing, the entropy of the time series of heartbeat intervals time series of people who are considered sedentary is reduced with respect to their resting value when people do moderate physical activity, in this case walking on a treadmill. On the other hand, for people who are active, that is to say, that they exercise regularly, the entropy values when doing physical activity are maintained or even increase when they walk at moderate speeds, it is necessary to greatly increase the speed of the treadmill to observe the decrease in entropy (Figure 9). This fact could be used to measure the physical condition of people; it seems important because it is currently known that regular physical activity of moderate intensity plays an important role in promoting good health and preventing diseases (Pate et al., 1995). For older adults, being regularly active is associated with better physical and even psychological health (McAuley and Rudolph, 1995). There is evidence indicating that, among older adults, low physical condition is a risk factor for functional impairment, and there is a positive effect of physical activity on functional limitations (Huang et al., 1998; Morey et al., 1998).

**FIGURE 10:**
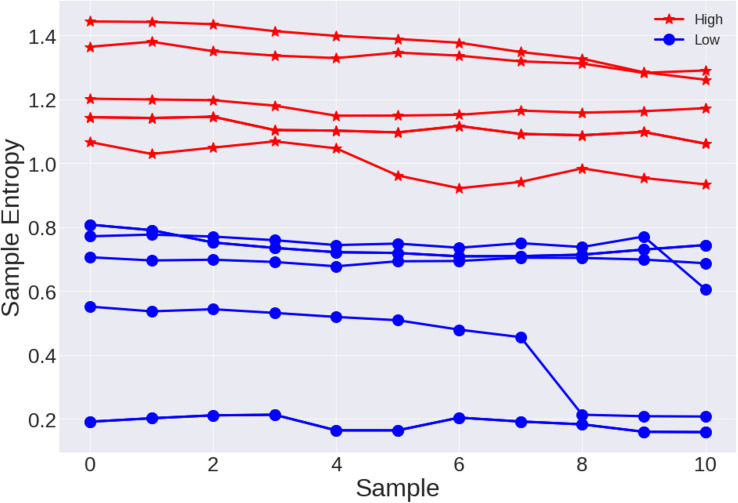
Five time series of active young subjects (red) and five time series of sedentary young subjects (blue) during the stress tests (4 miles per hour). The points represent SampEn values for subseries obtained from the time series with overlapping. For active subjects the average change in sampling entropy is –0.10, and for sedentary subjects is –0.11.

Finally, we know that when exercising, the heart rate increases, this is observed in Figure 3C, as well as a decrease in the RR times, which means that the heart rate tends to increase as the ST progresses. This led us to think that entropy could probably change not only due to the change in rest-exercise, but also due to fatigue. We arbitrarily took five series of active youth and five series of sedentary youth during the ST at 4 miles per hour and calculated the sampling entropy, in 1024-point subseries, then took another 1024-point subseries to the right but with an overlap with the first series of 100 points and so we continued to the right until the end of the test, that is to say, a windowing with overlap was made. The results are shown in Figure 10, as we can see, the entropy values for active youth are larger than those for sedentary. On average, there is a decrease in entropy in both cases, but such decreases are very small compared to the entropy changes associated with the change in rest-exercise.

Finally, we mention possible limitations of the present study: it is known that gender has a substantial effect on HRV. In a future work the size of the database will be increased to be able to make precise gender and age distinctions, because these influences need to be considered when performing HRV studies even if these influences only partly differ (Voss et al., 2015; Faes et al., 2019). It is also necessary to complement the study with the application of other non-linear techniques.

## Conclusion

It seems to be that beat-to-beat time series entropy analysis from continuous ECG recordings, while performing physical activity, may be effective in measuring fitness. We measured the entropy at rest and only for healthy subjects who do physical activity regularly a light increase in the entropy values was observed during the physical activity test, and a decrease in the entropy values was observed in those subjects with a sedentary lifestyle, this also happens for middle-aged adults. This work suggests that SampEn is a good measure of cardiovascular variability which can be related to physical condition and well-being. Although the entropy variations and the results obtained are reproduced for the SampEn, the ApEn, and the FuzzyEn, the sampling entropy is the best for quantifying the complexity of the HRV series.

The main finding of the study is the different behavior of entropy of the RR time series for sedentary and active people. While for sedentary people, entropy decreases during a ST compared to the resting state, for active people, entropy increases, indicating greater complexity in the latter case. The results are reproducible and different entropic measurements provide similar results. Although the sample size is relatively small, all the series are well characterized, they were pre-processed to ensure that the results are not altered by the length of the series, the presence of peaks or the presence of regions with extreme variability. Furthermore, supported by the IPAQ questionnaire, it was perfectly possible to characterize who of the participating subjects were sedentary and who were active subjects. It is important to emphasize that the entropy variations are significant despite the fact that in the STs the walk was carried out at very low speeds.

## Data Availability Statement

The datasets generated for this study are available on request to the corresponding author.

## Ethics Statement

Ethical review and approval was not required for the study on human participants in accordance with the local legislation and institutional requirements. The patients/participants provided their written informed consent to participate in this study.

## Author Contributions

AM-D contributed to the conception and design of the study, acquisition and analysis of the data, interpretation of the data, writing the article, critical revision of the manuscript, and funding acquisition. ES-M contributed to the interpretation of the data and writing the article. GG-C contributed to the acquisition of the data and writing of the article. All authors have read and approved the final manuscript.

## Conflict of Interest

The authors declare that the research was conducted in the absence of any commercial or financial relationships that could be construed as a potential conflict of interest.
